# No psychological effect of color context in a low level vision task

**DOI:** 10.12688/f1000research.2-247.v1

**Published:** 2013-11-15

**Authors:** Adam Pedley, Alex R Wade

**Affiliations:** 1 Department of Psychology, University of York, York, YO10 5DD, UK

## Abstract

**Background:** A remarkable series of recent papers have shown that colour can influence performance in cognitive tasks. In particular, they suggest that viewing a participant number printed in red ink or other red ancillary stimulus elements improves performance in tasks requiring local processing and impedes performance in tasks requiring global processing whilst the reverse is true for the colour blue. The tasks in these experiments require high level cognitive processing such as analogy solving or remote association tests and the chromatic effect on local vs. global processing is presumed to involve widespread activation of the autonomic nervous system. If this is the case, we might expect to see similar effects on all local vs. global task comparisons. To test this hypothesis, we asked whether chromatic cues also influence performance in tasks involving low level visual feature integration.

**Methods:** Subjects performed either local (contrast detection) or global (form detection) tasks on achromatic dynamic Glass pattern stimuli. Coloured instructions, target frames and fixation points were used to attempt to bias performance to different task types. Based on previous literature, we hypothesised that red cues would improve performance in the (local) contrast detection task but would impede performance in the (global) form detection task.

**Results:** A two-way, repeated measures, analysis of covariance (2×2 ANCOVA) with gender as a covariate, revealed no influence of colour on either task, *F*(1,29) = 0.289, *p* = 0.595, *partial η ^2^* = 0.002. Additional analysis revealed no significant differences in only the first attempts of the tasks or in the improvement in performance between trials.

**Discussion:** We conclude that motivational processes elicited by colour perception do not influence neuronal signal processing in the early visual system, in stark contrast to their putative effects on processing in higher areas.

## Introduction

There is now strong evidence that colour influence people’s performance on cognitive tasks. These effects are often demonstrated by pre-cuing a subject in a subtle manner (for example, by changing the colour of the ink or cover page in a written examination) and measuring performance in the exam as a function of cue colour.

These effects appear to be remarkably robust. For example, Elliot, Maier, Moller, Friedman & Meinhardt ^1^ showed that subjects performing a series of demanding cognitive tests performed worse when they were pre-cued with the colour red than when they were cued with control colours such as green, blue or grey. This effect was found across a wide range of cue conditions, exposure times, subjects, environments and even across two continents ^1^. Similar effects have been demonstrated in a series of other experiments. Mehta and Zhu ^2^ demonstrated that blue and red pre-cues similar to those described above influenced performance in tasks. In particular, blue cues led to better performance in creative-oriented, divergent tasks whilst red cues facilitated superior performance in detail-oriented, convergent tasks.

The effect can even be driven by colour language. For example, in a series of experiments Lichtenfeld, Maier, Elliot & Pekrun ^3^ showed that processing the *word* 'red' significantly impaired performance on both analogy and numeric IQ tasks. The most striking aspect of this research is the subtlety with which the colour manipulation took place. In one experiment in Germany, participants were simply assigned to either the 'red' (‘rot’) or 'place' (‘ort’) group without a visual colour exemplar. In another experiment the colour manipulation took the form of potential colour words in answers to a multiple choice-question. The effect has also been found when the colour stimulus was entirely incidental to the task. In one experiment the colour manipulation was the copyright label on a cover which read; 'Hogreffe Series of Tests' followed by either the word 'Red' or 'Grey'. Participants were not instructed to read the text but were required to wait with the page open until instructed to start. It seems that in these experiments only the neural evocation of the concept of a colour is required to influence performance.

These results are now commonly framed within a theory known as the ‘Colour-in-Context’ (CIC) theory ^4^. This formalizes the idea that colour carries semantic messages but that the behaviour resulting from these messages depends on context. In an ‘achievement context’, for example, many researchers believe that the colour red initiates avoidance behaviours through low-level physiological priming mechanisms. The neural correlates of this avoidance behaviour were identified in the Elliot *et al.* work ^1^ using electroencephalography (EEG). Conversely, several researchers have proposed that, in an achievement context, the colour blue initiates approach behaviours ^2^.

Maier, Elliot & Lichtenfeld ^5^ proposed that approach/avoidance behaviours can also be presented within a global/local processing framework. Specifically, it has been suggested that the colour red initiates a focusing or constricting of the attentional spotlight that facilitates the processing of local features while, the colour blue has the opposite effect, encouraging ‘big picture’ or ‘global’ feature processing.

The concepts of local and global processing are not defined rigorously in the context of the high-level cognitive tasks commonly used in the literature. In this paper, we examine claims of chromatically-cued task performance using a classic low-level visual paradigm that contains both local and global features. In all experiments, the nature of the psychophysical stimulus was constant and we examined the effect of ancillary chromatic features in a simple two-by-two factorial design. Specifically, we varied the colour of the fixation cross, instructions and target boundaries. We then examined the effects of these changes on performance in one of two tasks: global or local feature detection. Subjects were placed in an achievement context by encouraging a sense of competitiveness and instructing them that ‘success’ on the task would lead to further opportunities for (paid) research participation.

Despite the use of strong chromatic cues and an achievement context, we found no effect of cue colour on performance. Specifically, neither global nor local feature detection thresholds showed any dependence on cue colour. We also examined the effect of cue colour on learning rates and, similarly, found no effect. We discuss these findings with reference to both low-level visual processing and the CIC theory.

## Methods

### Subjects

Thirty native English speaking subjects (21 ± 2.9 years old, 9 male) were recruited and tested at the University of York, UK. All subjects had self reported normal or corrected to normal vision. Colour vision was assessed using a pseudo-Isochromatic Ishihara plate colour vision test ^6^. One subject could not complete the colour vision test and was therefore omitted from the study. One other subject was unable to complete the task to a satisfactory standard (1300% increase on one standard deviation (SD) in the local task, 679% increase on SD in the global task) and was also omitted from the study. Informed written consent was obtained from all subjects.

### Stimuli

We used dynamic Glass patterns ^7, 8^ to generate stimuli with both local and global components. Glass patterns ^7^ have long been used to investigate global and local feature processing ^9^. They are formed by placing two slightly shifted copies of an identical random dot pattern on top of each other, generating oriented pairs of dots termed ‘dipoles’. The type of transformation defines both the orientation and size of the dipoles and in turn, defines the overall global percept. Critically, the local statistics of the dipoles (for example, contrast and dot size) can be varied without affecting the global percept.

When individual Glass patterns are presented for long durations, they give rise to a static global percept of structure. However, when individual dipoles are replaced and updated rapidly they convey not only form but also a sense of implicit motion. This motion is perceived despite the fact that the position of the updated dipoles is uncorrelated from frame-to-frame ^10^. Such configurations of sequential brief presentations are termed 'dynamic Glass patterns' (dGP). Ross, Badcock & Hayes ^8^ proposed that the implicit motion generated by dGPs is a result of the form and motion aspects of vision combining. In effect, the circular form of the Glass pattern imposes structure to the otherwise random 'motion' of updated dipoles.

Glass patterns, and variations thereof, have been used to reveal the underlying physiological correlates of global form perception and it is now clear that different Glass pattern discrimination tasks (local vs. global) engage different neuronal populations ^11^. Several models have been proposed that describe the hierarchical process by which Glass patterns are perceived. Wilson & Wilkinson ^12^ proposed that the early local stage of filtering is facilitated by a selection of oriented spatial filters. When these are combined at a later stage the signals from these filters combine to form a global percept. Because of their simplicity and clear long- and short-range structure, Glass patterns are ideal stimuli for studying issues relating to global and local visual processing.

### The present study

In the colour-cognition literature that motivated this study, the presence of a red cue has been shown to facilitate local processing and impede global processing, whilst a blue cue has been shown to have the opposite effect ^2, 4^. Therefore, if we introduce colour priming cues into Glass pattern threshold experiments we expect to see differences in the performance of tasks requiring either local or global processing. In a red cue condition we would expect the local processing to improve (allowing subjects to make more accurate judgements about low-level features of the individual dipole elements) but the global perception of form (for example, the ability to distinguish structured dipole fields from unstructured ones) should be impeded. In a blue cue condition the reverse would be expected.

We measured local and global processing in two separate tasks. The global task was the detection of concentric dGP form against a backdrop of random noise. Performance in this task was expressed as the smallest global coherence level that could be detected reliably. The local task required subjects to detect changes in the contrast of the dipoles and performance in this task was expressed as a contrast discrimination threshold. The two conditions were orthogonal in the sense that small changes in contrast have no effect on the detectability of Glass pattern coherence and the presence or absence of global organization has no effect on subject’s ability to perform local contrast modulation tasks. We also note that the two tasks truly require global and local strategies: it is not possible to reliably distinguish structured from unstructured fields based on information from low-level ‘local’ receptive fields ^13^ and because we altered contrast (rather than luminance), pooled responses from very large ‘global’ receptive fields are uninformative about local dipole features.

To assess the effect of chromatic cue on global and local visual processing we introduced a set of chromatic stimulus components (fixation boxes, textual instructions) that could be either red or blue. Based on the existing literature regarding chromatic influences on neural processing we hypothesised that:

1. Red cues would facilitate local processing which would improve the ability to detect changes in contrast but impede the ability to detect dGP form.

2. Blue cues would have the opposite effect: enabling global processing and improving the ability to detect dGP form but impairing the ability to detect local contrast changes and therefore increasing thresholds on the contrast modulation task.

### Design

The experiment used a 2×2 repeated measures design. The independent variables were the colour manipulation (red vs. blue) and the type of task (local vs. global). The dependant measure was the detection threshold level of the task.

## Materials

**Figure 1.**
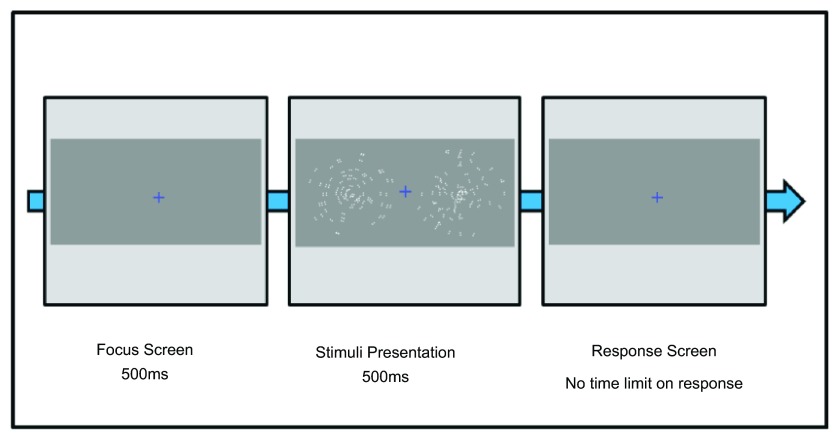
Pictorial representation of the dynamic Glass pattern (dGP) experiment trial.

Experiments were designed using Psykinematix Visual Psychophysics 1.4 software ^14^. The experiments consisted of two squares (6.5° radius, 3.0 point line width, no fill) presented horizontally at 5° of eccentricity. Within each square was a concentric dGP made up of 65 dipoles each with a spatial jitter of 0.25° and a lifetime of 0.005ms before being replaced. Concentric dGPs were used as stimuli as previous research has shown that the visual system is most sensitive to detecting these patterns ^15^. Each trial lasted 500ms before a response was required. The next trial started after a response was given (see Figure 1).

We used a two-alternative forced-choice procedure, presented spatially, in which participants were required to indicate, using a keyboard, which square contained either dGP form (global task) or dipoles with higher levels of contrast (local task). Feedback for correct/incorrect responses was given through auditory signals of high and low pitch respectively. Both coherence and contrast were controlled by a Bayesian adaptive threshold procedure ^16^ to determine 75% discrimination thresholds.

Stimuli were presented on a 20" cathode ray tube (CRT) monitor (Sony GDM-20E21, with a resolution of 1024×768 at 100Hz) driven by an ATI Radeon HD 5770 graphics card on an Apple Mac Pro (OS X 10.6.8). The display, with a mean luminance of 42cd/m ^2^ was calibrated prior to data collection using a ColourVision Spyder4Pro colour calibration unit and the Psykinematix Visual Psychophysics software. Calibration of the display was verified using a fibre-optic based spectrophotometer (USB2000, OceanOptics, Fl) which was itself calibrated against a NIST-traceable source. The display was situated in a dark, quiet room and subjects sat 80cm from the display.

Colours were defined in Macleod-Boynton/DKL colour space ^17, 18^ by specifying individual cone contrasts relative to a mean-grey background. In all instances of colour presentation the colours were either red (as defined by LMS cone contrast levels of L = -0.508, M = -0.826, S = 1.00, contrast = 39.12%) or blue (L = -0.920, M = -0.749, S = -1.00, contrast = 74.71%). These coordinates were constrained by several criteria. First, our chromatic stimuli were chosen to represent ‘pure’ exemplars of red and blue hues. This type of ‘unique hue’ setting varies slightly from person to person but when queried, our own subjects agreed on the canonical names of these hues unanimously. We used *contrast* levels (computed relative to a mean gray background) to allow for good replicability and stability of our stimuli: in particular light adaptation levels are not well defined when colours are presented on a CRT against a black background. Finally, to maximize the potential psychological effects of our cues, we used highly saturated colours that were, necessarily, at the limit of the monitor gamut. These constraints meant that our chromatic cues were not isoluminant: the luminance contrast (as computed from the RMS cone coordinates) of the blue stimulus components was approximately twice that of the red components. This confound was unavoidable given the other constraints. However, in both cases, the luminance contrast was well above detection thresholds so that the cues were highly visible (and therefore the instructions, for example), were legible on the basis of luminance alone.

Colour cuing was achieved in three ways. First, each experiment had instructions presented in coloured text that were displayed prior to the experiment starting. These instructions were presented in Arial Bold font, 36pt and were displayed until the subject pressed a key to initiate the experiment (approx 10s). Secondly, the fixation cross in the centre of the screen was either red or blue. Finally, the lines of the squares in which the glass patterns were presented were coloured either red or blue. All colour cues were consistent within an experiment. This resulted in four separate experiments; a red and blue version of both the local and global experiments.

### Procedure

Subjects were shown an example of each experiment (devoid of colour manipulation) and were required to read a set of pre-experiment instructions, printed black ink on white paper. Each subject then completed each of the four experiments twice (eight experiment blocks of eighty trials giving 640 trials in total). The order of the blocks was varied randomly across subjects. After the experiment had finished the subjects completed a verbal de-briefing task to determine if subjects were aware of the colour manipulation. The de-briefing task comprised of nine questions and refers to the purpose of the experiment and the colours employed within.

To ensure that participants performed the task within a strong achievement context they were informed that failure to attain a certain score would result in them not being able to continue to the second part of the experiment, a four question online survey. After the experiment, all participants were informed that they successfully completed the task and were sent the online survey to complete. The experiment was approved by the University of York ethics committee.

### Statistical analyses

All statistical analyses were run using IBM SPSS ver. 20.0. Data screening involving the calculation of skewness and kurtosis z-scores, Kolmogorov-Smirnov test and Shapiro-Wilk test confirmed normality of the data. Dependent t-tests were conducted to assess if colour cues (red vs. blue) influenced performance within tasks (local vs. global). A two-way repeated measures analysis of covariance (ANCOVA) was conducted to assess if there were any interactions between the colour cues and the type of task with gender factored as a covariate. All inferential analyses used a *p* value cut off of <.05 to determine significance.

## Results

### Detection thresholds

We first asked if the cue colour influenced performance in either task. Specifically, we wanted to determine if a red cue improved performance (reduced discrimination thresholds) in the local task and impeded performance in the global task and if the opposite effect occurred for the blue cue condition. Unless otherwise stated the values used were from the second trials.

**Figure 2.**
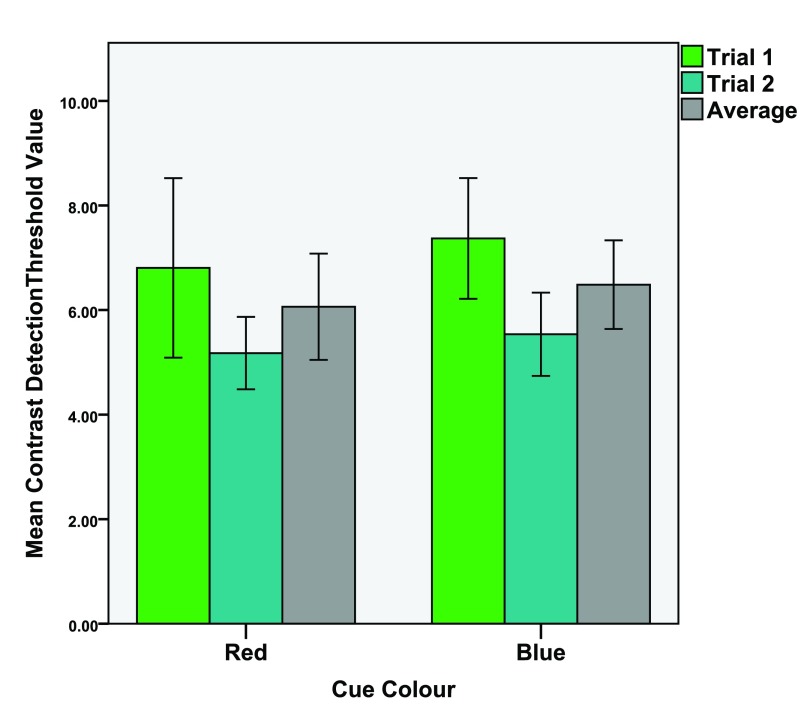
Mean detection threshold values for the contrast (local) task. Mean values of the 1st and 2nd trials as well as the average value of both trials. Error bars indicate the 95% confidence interval.

A dependant t-test was conducted (colour condition red vs. blue) on local task detection thresholds (M = 5.36, SD = 1.99). The analysis revealed no significant differences between the red (M = 5.18, SD = 1.86) and blue (M = 5.54, SD = 2.13) colour conditions, *t*(29) = -1.184, *p* = 0.246, *d* = 0.180. See Figure 2 for means of both trials and their average value.

**Figure 3.**
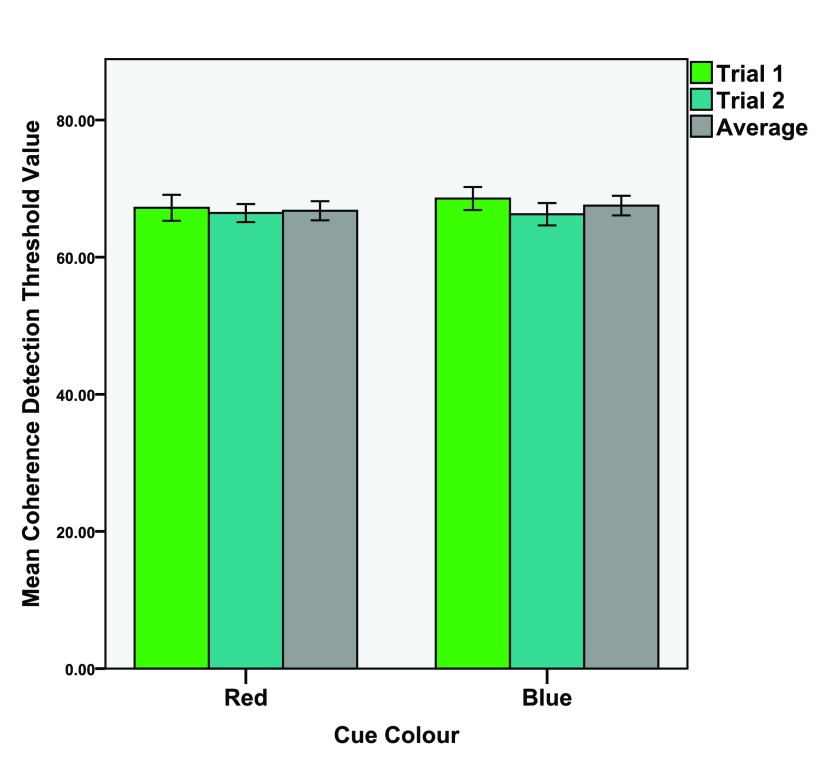
Mean detection threshold values for the coherence (global) task. Mean values of the 1st and 2nd trials as well as the average value of both trials. Error bars indicate the 95% confidence interval.

A dependant t-test was conducted (colour condition red vs. blue) on global task detection thresholds (M = 66.35, SD = 3.93). The analysis revealed no significant differences between the red (M = 66.43, SD = 3.58) and blue (M = 66.26, SD = 4.37) colour conditions, *t*(29) = 0.218, *p* = 0.829, *d* = 0.043. See Figure 3 for means of both trials and their average value.

We also asked if there were any interactions between the two types of task (local and global) and the two colour conditions (red and blue). As gender has been claimed to affect global vs. local visual processing ^19^ it was included as a covariate in this analysis. We performed a 2-way repeated measures analysis of covariance (ANCOVA) and asked if there was a main effect of colour on task, with gender as a covariate, and if there were any interactions between gender, cue colour and task. None of these effects were significant. We found no effect of colour on task performance, *F*(1,28) = 0.289, *p* = 0.595, *partial η ^2^* = 0.002, no significant interaction between task and colour, *F*(1,28) = 0.081, *p* = 0.77, *partial η ^2^* = 0.002 and we found no significant interaction between gender and task performance, *F*(1,28) = 0.737, *p* = 0.398, *partial η ^2^* = 0.013. Finally, this analysis revealed no significant three way interactions of task, colour and gender, *F*(1,28) = .014, *p* = 0.905, *partial η ^2^* < 0.001.

### Examining differences in novel tasks

The majority of studies in the literature to date have used a between-subjects design in which subjects completed only one of the tasks ^1, 2^. It is possible that the absence of significant effects in our own study could be a result of motivational processes being elicited only when first completing a novel task. To control for this, we performed an analysis which only considered each subject's first trial in the experiment block. Significant results would indicate that the influence of cue colour only impacts performance when a task is novel and the effect diminishes through practice or habituation.

**Figure 4.**
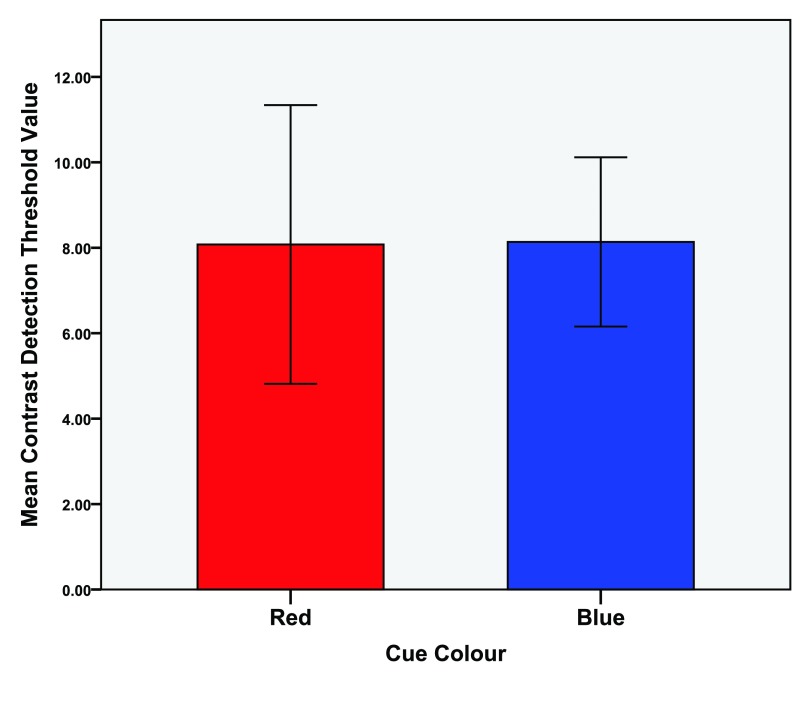
Mean detection threshold values for the first attempts at the contrast (local) task. Error bars indicate the 95% confidence interval.

An independent t-test was conducted (colour condition red vs. blue) of the first attempts of both the local and global task (N = 15). The local task analysis (M = 8.107, SD = 4.79) revealed no significant differences between the red (M = 8.08, SD = 5.89) and blue (M = 8.14, SD = 3.58) colour conditions, *t*(14) = -0.041, *p* = 0.968, *d* = 0.012. See Figure 4 for means of the first attempts at the local task as a function of colour.

**Figure 5.**
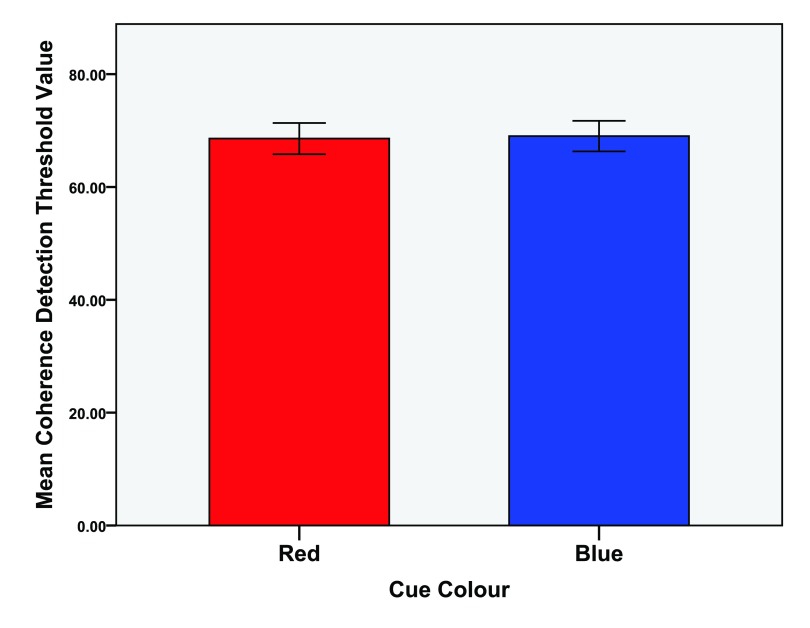
Mean detection threshold values for the first attempts at the coherence (global) task. Error bars indicate the 95% confidence interval.

The global task analysis (M = 68.80, SD = 4.87) revealed no significant differences between the red (M = 68.58, SD = 5.00) and blue (M = 69.02, SD = 4.90) colour conditions, *t*(14) = -0.233, *p* = 0.819, *d* = 0.088. See Figure 5 for means of the first attempts at the global task as a function of colour.

### The effect of colour on performance improvement

Most subjects achieved slightly lower thresholds on their second trials compared to the first trials. Although no effect of colour was detectable in the thresholds of either the first or second trials examined separately, we wondered if colour might still be influencing the rate of improvement.

We tested this by comparing the first trials to the second trials of the experiments (see Figure 2 and Figure 3). Dependant t-tests were conducted (1st trial vs. 2nd trial) of the four conditions. The results were; *Red local task*; 1st trial (M = 6.81, SD = 4.60), 2nd trial (M = 5.18, SD = 1.86), *t*(29) = 1.964, *p* = 0.030, *d* = 0.462. *Blue local task*; 1st trial (M = 7.37, SD = 3.09), 2nd trial (M = 5.54, SD = 2.13), *t*(29) = 3.56, *p* < 0.001, *d* = 0.693. *Red global task*; 1st trial (M = 67.20, SD = 5.06), 2nd trial (M = 66.43, SD = 3.58), *t*(29) = 0.944, *p* = 0.163, *d* = 0.190. *Blue global task*; 1st trial (M = 68.54, SD = 4.50), 2nd trial (M = 66.26, SD = 4.37), *t*(29) = 2.748, *p* = 0.05, *d* = 0.514.

Three of the four comparisons revealed significant differences. However, there was no significant improvement between the first and second trial in the red global task. To further examine these differences in performance, we asked if the colour condition influenced the magnitude of the shift in performance between the first and second task. Any differences here could indicate that colour stimuli influence the learning curve of local or global tasks.

**Figure 6.**
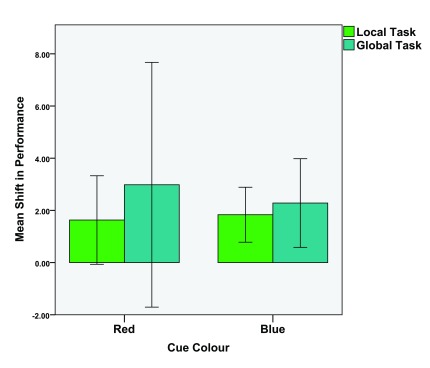
Mean shift-in-performance values of both the contrast (local) and coherence (global) tasks. Error bars indicate the 95% confidence interval. N.B., the error bars for the red cue condition straddle zero as some participants exhibited a negative shift-in-performance.

To visualize these effects, we calculated a 'shift-in-performance' (SIP) parameter by subtracting the threshold of the second trial from the threshold of the first trial. Two dependant t-tests were conducted (colour condition red vs. blue) on the SIP values of both the local tasks (M = 1.73, SD = 3.68) and global tasks (M = 2.63, SD = 8.56). The local task analysis revealed no significant differences between the red (M = 1.63, SD = 4.55) and blue (M = 1.83, SD = 2.82), colour conditions, *t*(29) = -0.205, *p* = 0.420, *d* = 0.053. The global task analysis revealed no significant differences between the red (M = 2.98, SD = 12.56) and blue (M = 2.28, SD = 4.55) colour conditions, *t*(29) = 0.319, *p* = 0.376, *d* = 0.074. See Figure 6 for mean SIP values of the local and global tasks as a function of colour.

### Relationship between performances in local and global tasks

Previous research has shown that individuals who perform better in global tasks often perform worse in local tasks and vice versa ^20^. A Pearson's correlation was conducted (colour conditions red vs. blue) which revealed no significant correlations of either the red condition local/global tasks, *r* = 0.235, *p* = 0.469, or the blue condition local/global tasks, *r* = 0.216, *p* = 0.285. We found no relationship, positive or negative, between the performance in the two tasks.

### Participants' awareness of colour manipulation

We were not subtle in our presentation of colour stimuli. We manipulated the highly-saturated colour of the instructions, fixation cross and on-screen stimuli. Given this overt colour manipulation and in line with previous research ^1, 3^ we assessed, through a verbal funnelling task, the subjects' awareness of the colour manipulation and the purpose of the experiment. No participants guessed the aims of the experiment. To assess the awareness of colour in the experiment we analysed responses from two questions in the verbal funnelling task;

*'Can you name the colour of the fixation cross?'*

*'Can you name the colour of the instructions at the beginning of each experiment?'*

Results showed that 50% of subjects (N = 15) were aware of the colour manipulation and could report both presented colours. 20% (N = 6) were able to name one of the presented colours. 30% of subjects (N = 9) were unable to recall either of the presented colours. Of these 9 subjects, 8 reported seeing colours that were not presented; black, white and yellow. These results indicate that half of the subjects could name the colours in the experiment but this did not reveal the aims or impact on the validity of the study.

Raw data of threshold values of recognising either contrast changes in dipoles (local task) or the presence of coherent motion in glass patterns (global task) CSV: Values are given for each task (local and global) and each colour (red and blue) across each trial (first and second). The mean values for each participant are also given. In total 12 values for each participant are given. Gender of Participant - '1' = male, '2' = female.
Instructions PDF: Instruction scripts given to participants before the task.
De-funnelling PDF: De-funneling task used to assess whether the participants were aware of colour manipulation during the experiment. Administered verbally.

## Discussion

Our primary finding is a negative result. Contrary to the existing literature, we found no effect of colour on performance in low level visual tasks set in an achievement context. We found no significant differences in the performance of either the local or global tasks as a function of colour cues. We found no significant differences in the first attempts of either task or in the improvement of performance between trials. Subjects' scores improved significantly between trials in most experiments, presumably due to training effects. However further analysis revealed no relationship between training-dependent improvement and colour. As in previous work (see Elliot and colleagues), the significance of the overt colour manipulation that took place was undetected by participants.

Earlier studies have observed strong effects of colour on performance in global tasks such as anagrams, analogies, number sequence tasks ^1, 3^, ‘creative use’ tasks ^2, 21^ and in ostensibly local tasks such as proof reading and memorization ^2^. While negative results can arise from trivial causes such as poorly-designed experiments or insufficient statistical power, we do not believe these criticisms apply to the current study. We tested a large number of subjects from a relatively homogeneous population, our experimental design was a direct extrapolation of those used in previous studies and the tasks and our stimuli were well-controlled and commonly used in the vision science literature to examine precisely this type of local vs global processing ^11^.

Previous publications detail at least 19 experiments (across 5 publications) demonstrating that colour influences performance with some reference to the theoretical framework of the CIC ^1– 3, 5, 21^. The effects have been documented across experiments using various exposure times (2s, 5s and constant exposure) and across a wide array of colour presentation techniques (screen background colour, ink and paper colour or a lexical term). The experiments have been conducted in various institutions across Northern America and Europe in both laboratories and classrooms. We argue not that our work contradicts these findings but rather we have identified an area of task performance that remains unaffected by colour cues. In short, we propose that the motivational processes evoked by colour cues in achievement contexts do not influence low level visual perception.

Where in the visual system are the global and local aspects of our task performed? While local contrast judgements can be supported by short-range V1 receptive fields, detecting and discriminating dynamic Glass patterns requires long-range integrative mechanisms. Wilson, Wilkinson & Assad ^22^ proposed that the perception of Glass patterns occurs through the ventral stream within the neurophysiological account of visual perception. Initial filtering of the dipoles occurs in V1, followed by a second filtering stage in V2. The global spatial pooling of these local features occurs in V4. These claims are supported by empirical evidence. Smith, Bair & Movshon ^13^ demonstrated that the size, structure and performance of the receptive fields in V1/V2 neurons are particularly well suited to analyse the orientation of dipoles. Separate experiments have demonstrated that V1 ^13^ and V2 cells ^23^ are largely unable to detect the global form of glass patterns. Where modulation of V1 activity in response to global pattern changes is observed, it is most likely due to feedback from higher areas ^24^.

On average, V4 neurons have receptive fields 5–7 times larger than those in V1/V2 ^25^ meaning they are much better suited to integrate across the visual field and detect the global form of Glass patterns. Tse *et al.* ^26^, examined how V1/V2/V4 neurons responded to different glass patterns in macaques. They found that whilst the response of the V4 neurons varied between different Glass patterns, the responses of V1/V2 cells remained stable regardless of the type of Glass pattern presented. This suggests that global form information is extracted at a later stage than local features such as dipole contrast.

In short, the local features of dGPs are processed first in visual areas V1 and V2 before the formation of the global percept in ventral and lateral areas such as V4 and the LOC. Both these processes take place before visual information reaches the higher level areas of the ventral stream in the inferior temporal (IT) lobe where semantic value is added to visual information. We therefore propose that while general motivational processes evoked by colour perception do influence performance in some tasks that require high level cognition (i.e. anagram task, creative uses task) they do not influence neuronal signal processing in the early visual system.

We note that there are well-established examples of higher-level mechanisms affecting responses and psychophysical performance based on low-level visual areas. For example, attention to selective features of a stimulus (for example, its motion or colour) generate activations in cortical regions that are sensitive to those features ^27, 28^ and top-down attentional mechanisms have been found to affect the sensitivity, tuning and baseline activity of neurons as early as the LGN and V1 ^29– 34^. However, it appears that chromatic context does not generate a form of top-down modulation that falls into one of these classes.

This study has practical implications for psychophysical experiments assessing low level vision. The existing body of work ^2, 4^ would indicate it is vital that the use of colour in experiments is monitored carefully so as to not bias performance through motivational processes. This work demonstrates that in tasks requiring processing in the early visual system, such as contrast, motion and dGP form detection, no such consideration is required.
