# Supplementary material for: No psychological effect of color context in a low level vision task
Source: F1000Res. 2013 Nov 15;2:247. [Version 1] doi: 10.12688/f1000research.2-247.v1 (PMC4097361; doi:10.12688/f1000research.2-247.v1)
Supplement: Raw data of threshold values of recognising either contrast changes in dipoles (local task) or the presence of coherent motion in glass patterns (global task) — CSV: Values are given for each task (local and global) and each colour (red and blue) across each trial (first and second). The mean values for each participant are also given. In total 12 values for each participant are given. Gender of Participant - '1' = male, '2' = female. Instructions PDF: Instruction scripts given to participants before the task. De-funnelling PDF: De-funneling task used to assess whether the participants were aware of colour manipulation during the experiment. Administered verbally. [file f1000research-2-2594-s0000.tgz › Instruction_scripts.pdf]

Instruction scripts for both experiments

Global task instructions

You will be shown two squares containing moving dots

One of the squares will contain more dots moving in a circular motion.

Keep your vision focussed on the cross in the centre of the screen at all times.

Indicate which square contains more circular moving dots.

Use the 'left arrow' for the left square and the 'right arrow' for the right square.

Correct responses are signalled with a 'high beep'.

Incorrect responses are signalled with a 'low beep'.

If you cannot tell the difference between the squares, please guess.

The test will run for 80 trials.

The first ten trials are treated as 'training' and do not count towards the final score.

There are 8 experiments. Each experiment will take 3 minutes.

If your overall score is above 75% you will be asked to fill out an online questionnaire at a later date.

If your overall score is below 75% you will not be able to continue with the experiment and your data will be removed.

Any questions?

You may start.

Local task instructions

You will be shown two squares containing moving dots

One of the squares will contain dots with higher contrast.

Keep your vision focussed on the cross in the centre of the screen at all times.

Indicate which square contains dots with higher contrast.

Use the 'left arrow' for the left square and the 'right arrow' for the right square.

Correct responses are signalled with a 'high beep'.

Incorrect responses are signalled with a 'low beep'.

If you cannot tell the difference between the squares, please guess.

The test will run for 80 trials.

The first ten trials are treated as 'training' and do not count towards the final score.

There are 8 experiments. Each experiment will take 3 minutes.

If your overall score is above 75% you will be asked to fill out an online questionnaire at a later date.

If your overall score is below 75% you will not be able to continue with the experiment and your data will be removed.

Any questions?

You may start.
