# Supplementary material for: No psychological effect of color context in a low level vision task
Source: F1000Res. 2013 Nov 15;2:247. [Version 1] doi: 10.12688/f1000research.2-247.v1 (PMC4097361; doi:10.12688/f1000research.2-247.v1)
Supplement: Raw data of threshold values of recognising either contrast changes in dipoles (local task) or the presence of coherent motion in glass patterns (global task) — CSV: Values are given for each task (local and global) and each colour (red and blue) across each trial (first and second). The mean values for each participant are also given. In total 12 values for each participant are given. Gender of Participant - '1' = male, '2' = female. Instructions PDF: Instruction scripts given to participants before the task. De-funnelling PDF: De-funneling task used to assess whether the participants were aware of colour manipulation during the experiment. Administered verbally. [file f1000research-2-2594-s0000.tgz › Defunnelling_task.pdf]

De-funnelling task

Study purpose questions

1. What do you think we were trying to test?

2. What do you think was changed throughout the experiments?

3. Do you think anything affected your performance in the tasks?

If colour is mentioned;

1. Do you have any idea of what the purpose of changing colour could be?

2. Do you think you performed differently under different colours?

'Awareness tests'

1. How many dots were moving in each square? (decoy question)

2. Can you name the colour of the fixation cross in the experiments?

3. Were the squares split left to right in every experiment? (decoy question)

4. Can you name the colour of the instructions?
