# Supplementary material for: No psychological effect of color context in a low level vision task
Source: F1000Res. 2013 Nov 15;2:247. [Version 1] doi: 10.12688/f1000research.2-247.v1 (PMC4097361; doi:10.12688/f1000research.2-247.v1)
Supplement: Peer review history

# Peer review: Referee response for version 1

**DOI:** 10.5256/f1000research.2594.r4429

**Published:** 2014-04-22

**Authors:** Arnold Wilkins

**Affiliations:** 1 Department of Psychology, University of Essex, Colchester, UK

In the investigation of the effects of colour on behaviour, as in the investigation of hypnosis, it is difficult to separate from other effects the influence of participants’ prior beliefs and expectations. These are culturally determined, and may play a role in influencing outcomes. Within contemporary culture there are widely held beliefs about the effects that colour may have on behaviour, and these have the potential to alter the participants’ motivation and attitudes towards any study involving colour. Attitudes and motivation are more likely to play a role in those cognitive tasks that have given rise to the Colour in Context theory, than in the low-level visual task used here in which performance is judged using two-alternative Psychophysics.

The current study appears to have been carefully designed and conducted, but perhaps slightly less carefully reported. I would have liked to have seen the stimuli described in greater detail. For example:

- What were the chromaticities of the red and blue colours used?
- It is unclear what made it impossible to match the luminances.
- Was the positioning of the square such that the centre was at 5 degrees eccentricity, or was it the nearest margin that was at 5 degrees eccentricity?
- Was the “radius” of the square measured horizontally or diagonally?
- I do not see how the lifetime of the dipoles could have been 0.005ms. Even if this is a typo and the lifetime was 0.005s, I am still incredulous because the screen refresh rate was 100Hz, so the frame duration was 0.01s and this is twice the supposed duration of the dipoles!

A t-test is not a dependant, even when it is a dependent t-test.

I would have liked to have seen some discussion of the possible role of colour in affecting the accommodative lag to the visual stimuli on display. There is data in the literature that could have been used to argue that the effects of colour on image blur would have been negligible (about 0.07 dioptres, at maximum), and in the event any effects of accommodation were minimal because colour had no effect on visual performance of either local or global tasks.

Given that about half the participants were aware of the colour manipulation and half were not, it might have been of interest to have separated the data for these two groups to see whether any effects of belief (“Colour in Context”) were more apparent in the group that had awareness. Indeed, in the literature on "Colour in Context", it would have been useful to have known what preconceptions regarding the effects of colour the participants brought to the tasks they were asked to perform.

I have read this submission. I believe that I have an appropriate level of expertise to confirm that it is of an acceptable scientific standard.

# Peer review: Referee response for version 1

**DOI:** 10.5256/f1000research.2594.r2457

**Published:** 2014-01-20

**Authors:** David Brainard

**Affiliations:** 1 Department of Psychology, University of Pennsylvania, Philadelphia, PA, USA

This is a well conducted study that examines whether cuing effects of color on 'local' and 'global' processing manifest with relatively low-level visual tasks. The authors find no such effects, with reasonably well-powered measurements. Indeed the measurements have sufficient power to reveal a learning effect within a subjects’ replications of the task. The error bars for the global task are particularly small. I have only minor suggestions:

1. Although certainly not for this paper, I would be interested in whether the present authors are able to obtain color cuing effects in an exact replication of some of the published studies that do obtain such effects. This might be a good project for a registered replication attempt.
2. The paper as it stands now is in excellent shape. If the authors choose to revise it, I think a quantitative expansion on the classification of their tasks as local and global would be helpful. For example is the spatial integration area known for each task, for stimuli like those employed by the authors? Direct measurements of the integration area would establish experimentally the locality/globality of each task in a satisfying manner.
3. Another analysis that could be considered would be to look at individual observer data. Do any observers show reliable cuing effects, and if so in what direction?  
4. *"... because we altered contrast (rather than luminance), pooled responses from very large ‘global’ receptive fields are uninformative about local dipole features."*  Although it is easy to believe that contrast discrimination is more local than identifying pattern structure, I don't understand this particular argument. It appears from Figure 1 that the pattern elements were all increments, so that changing contrast must also have changed luminance.  
5. Typo: “ *To assess the effect of chromatic cue”* should be changed to: “To assess the effect of chromatic cues”

I have read this submission. I believe that I have an appropriate level of expertise to confirm that it is of an acceptable scientific standard.
